# Spike Code Flow in Cultured Neuronal Networks

**DOI:** 10.1155/2016/7267691

**Published:** 2016-04-27

**Authors:** Shinichi Tamura, Yoshi Nishitani, Chie Hosokawa, Tomomitsu Miyoshi, Hajime Sawai, Takuya Kamimura, Yasushi Yagi, Yuko Mizuno-Matsumoto, Yen-Wei Chen

**Affiliations:** ^1^NBL Technovator Co., Ltd., 631 Shindachimakino, Sennan 590-0522, Japan; ^2^Department of Radiology, Graduate School of Medicine, Osaka University, Suita 565-0871, Japan; ^3^Biomedical Research Institute, AIST, Ikeda, Osaka 563-8577, Japan; ^4^Department of Integrative Physiology, Graduate School of Medicine, Osaka University, Suita 565-0871, Japan; ^5^College of Health and Human Sciences, Osaka Prefecture University, Habikino, Osaka 583-8555, Japan; ^6^ISIR, Osaka University, 8-1 Mihogaoka, Ibaraki City, Osaka 567-0047, Japan; ^7^Graduate School of Applied Informatics, University of Hyogo, Kobe 650-0047, Japan; ^8^College of Information Science and Engineering, Ritsumeikan University, Kusatsu 525-8577, Japan

## Abstract

We observed spike trains produced by one-shot electrical stimulation with 8 × 8 multielectrodes in cultured neuronal networks. Each electrode accepted spikes from several neurons. We extracted the short codes from spike trains and obtained a code spectrum with a nominal time accuracy of 1%. We then constructed code flow maps as movies of the electrode array to observe the code flow of “1101” and “1011,” which are typical pseudorandom sequence such as that we often encountered in a literature and our experiments. They seemed to flow from one electrode to the neighboring one and maintained their shape to some extent. To quantify the flow, we calculated the “maximum cross-correlations” among neighboring electrodes, to find the direction of maximum flow of the codes with lengths less than 8. Normalized maximum cross-correlations were almost constant irrespective of code. Furthermore, if the spike trains were shuffled in interval orders or in electrodes, they became significantly small. Thus, the analysis suggested that local codes of approximately constant shape propagated and conveyed information across the network. Hence, the codes can serve as visible and trackable marks of propagating spike waves as well as evaluating information flow in the neuronal network.

## 1. Introduction

Spike trains can be observed in a neuronal network. They show various aspects of neurons participating in the network. It is difficult, however, to determine how the spikes are coded. Furthermore, neurons work slowly and unreliably compared with artificial transistors, presenting a mystery of how a neuronal network can work intelligently and reliably.

The present methods of spike-coding analyses of neuronal networks are as follows.

*(A) Spike-Coding Metrics*. To analyze spike trains, the metrics between spike trains have been proposed on the basis of the alignment of the distances and convolution metrics, including traditional rate coding [1]. However, the coding scheme of neurons has not been solved by this method. 

*(B) Spatiotemporal Coding*. The extension of signals to multidimensional space permits the examination of many spatiotemporal patterns in artificial and natural neural networks [2–5]. In the visual system, in particular, directional receptive fields, which are similar to those observed in mammalian simple cells, emerge on the basis of a minimum information criterion [6], and an independent component analysis [7] of natural and facial images, which is a spatially independent basis function, is derived by self-organization. The receptive fields of the visual system are obtained by the self-organization of the neural circuit with mutual inhibition so that only spatially independent components are produced [8]. Thus, it is reasonable to seek the temporally independent components of information representation in the brain as a pair of spatially independent components or seek the spatiotemporal information representation and communication coding scheme. 

*(C) Synchronous Action Model*. Synchronous actions of* in vivo* neuronal networks are often observed. Abeles [9, 10] proposed a synfire chain, which is a model of neuron groups firing in a volley. Furthermore, Izhikevich [11] has proposed a model of neuron networks generating rhythmic actions. These are simulation models that explain the global actions of neural networks. Also Perc and Zhang et al. [12, 13] analyzed and showed stable and unstable waves sometimes mixing in neural network owing to characteristic changes of neurons.

*(D) Pseudorandom Code Analysis*. From the viewpoint of the coding scheme of spike trains, we showed that M-sequence-related codes are detected significantly more often than those from time-shuffled trains [14]. These may contribute to communication between neurons from an analogy of artificial communication systems.

In this study, to clarify the spike-coding mechanism, we first analyzed the spike trains of cultured neural networks by examining the code of a multielectrode array. Combination of relatively homogeneous cultured neuronal network and multielectrode recording will show relatively fundamental characteristics of coding scheme in neuronal network. Next, we visualized the flow of codes that were composed of spike sequences. We further quantified the flow of the codes that may reflect the flow of information in the neural network.

## 2. Code Spectrum of a Cultured Neural Network

**Figure 1:**
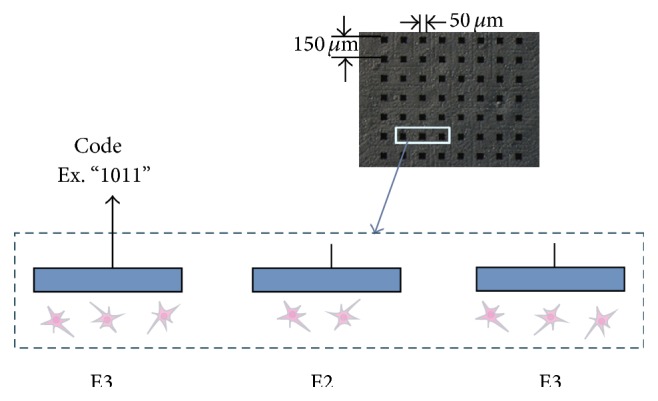
(Upper) Micrograph of cultured hippocampal neurons in a microelectrode array. Black rectangles indicate electrodes. (Lower) Illustration of a vertical section. Each electrode catches spikes from several neurons. We can observe spike trains containing code such as “1011.” Each bit (“1” or “0”) is considered from different neuron for short time length (short bit width) code, since it takes more time for the same neuron to fire twice than the refractory period.

The cell cultures of hippocampal neurons were dissected from 18-day-old Wistar rat embryos and implanted on microelectrode array dishes (MED-P515A, Alpha MED Scientific Inc., Kadoma, Osaka, Japan) with 8 × 8 planar microelectrodes as shown in Figure 1 [14]. In the present study, the same raster plot data were used as [15], which are composed of 0.1 ms bins.

**Figure 2:**
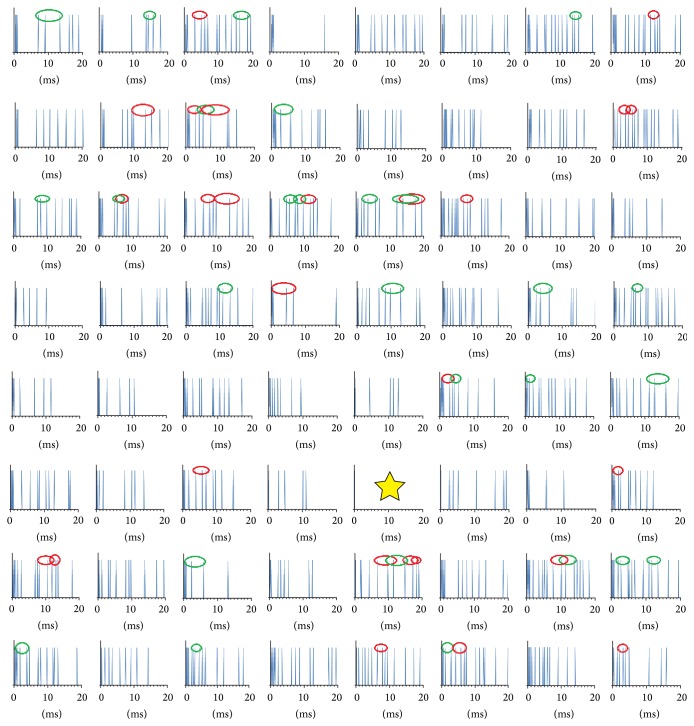
Spike trains on 8 × 8 multielectrodes between 0 and 18 ms (horizontal axis) after the stimulation pulse is given at time 0 from the electrode marked with a star. The red ellipse shows code “1011,” and the green ellipse shows “1101,” with each having a bit width more than 0.6 ms.

Because we did not sort the spikes, the spike train from each electrode may be composed of spikes from several neuronal cells. From these spike trains, we confirmed that the M-sequence family occurred significantly more often than by chance [14]. In Figure 2, we show the “1101” and “1011” detected codes of Sample A as the simplest code pair with 1% nominal time accuracy on the 8 × 8 electrode arrangement up to 18 ms after the neurons were stimulated. Codes “1101” and “1011” are a core part of the reversal sequences “1101000” and “1011000” of the representative M-sequences of “0010111” and “0100111,” respectively.

**Figure 3:**
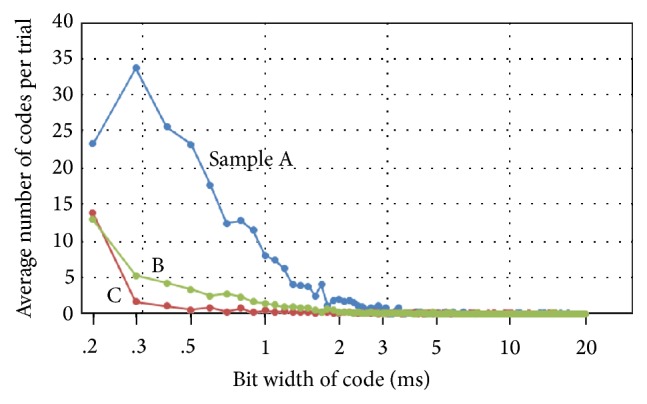
Average number of “1011” and “1101” codes that were observed per trial from 64 electrodes during the first 200 ms after stimulation versus the bit width of the code. We can see the bit width of the codes detected is mainly less than 2-3 ms.

**Figure 4:**
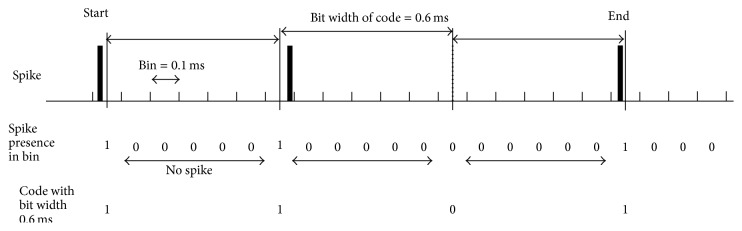
Code “1101” detected with bit width 0.6 ms.

Expanding this to 200 ms after stimulation, we obtained the histogram in Figure 3 of the sum of the “1011” and “1101” detected codes per trial among the 9 trials of Sample A, the 23 trials of Sample B, and the 26 trials of Sample C, in which we decoded spike trains from 63 electrodes excluding one stimulation electrode with various bit widths within 0.2–20 ms and 1% time accuracy for the inner bit of code. Here, a bit width is the time interval between “0” (no spike) or “1” (spike exists) and the next “0” or “1” in an assumed code (“1011” or “1101”) being detected as shown in Figure 4. First, two “1”s from the same electrode that were adequately separated in time were assumed to be the beginning and the ending “1”s of a code, and then if only one (and no other) inner “1” corresponding to the inner bit (“1”) of the code was detected with a 1% time accuracy between the beginning and ending “1”s, we decide that code “1101” (or “1011”) was detected. If there is no “1” between the assumed beginning and the ending “1”s of a code, it is decided not to be a code. Figure 3 indicates that the numbers for 0.2–0.5 ms are large. Because a bit width of 0.5 ms is less than the synaptic delay, these codes may be mainly composed by independent processes or appear by chance (e.g., code overlapping as seen in Figure 2), which does not help circuit analysis around the electrode. Furthermore, those greater than 2 ms are far less frequent than those of 0.6–2 ms. Therefore, we mainly investigated the codes with bit widths of 0.6–2.0 ms in the following analysis. Because the refractory period of neuronal cells is greater than 2.0 ms, the codes may be composed of spikes from several neuronal cells.

**Figure 5:**
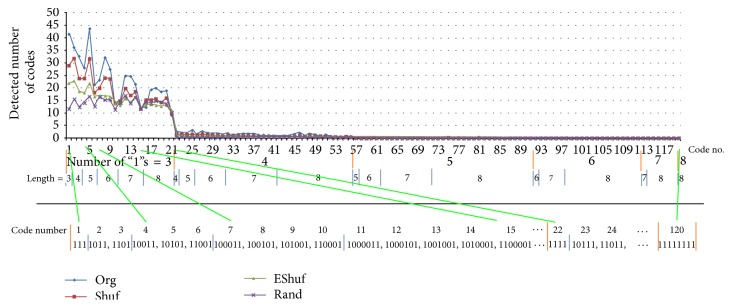
Spectrum of the detected codes with bit widths of 0.6–2.0 ms. We can see that the major component of the code spectrum is that of three bits (code number 1-21). Four bits or more codes (code number 22-120) are far less than that. Random train has flat spectrum within three-bit codes while the original train has its own shape. Interval shuffled and electrode shuffled spike trains show intermediate spectrum profiles.

Figure 5 shows a code spectrum from the 63 electrodes as an average of the 9 trials of Sample A, in which the targeted and detected codes (sequences) were those having binary “1”s at both ends of the code and more than three “1”s, including both ends, with lengths less than 8 bits and the sequence between the terminal “1”s was an incremental binary number as [“111”, “1011”, “1101”, “1111”, “10011”,…, “11111111”]. Since the number of “1”s in each code is inconsistent, the order of the codes was sorted by the number of “1”s in the code. The total number of codes under investigation was 120. The length of the train data was 200 ms [2,000 data points/(electrode × trial)], which was sampled with a 0.1 ms bin width, and the number of spikes (“1”s) on an electrode was an average per trial of 23.2 ± 9.1 in Sample A. The interval shuffled trains (Shuf), the electrode shuffled trains (EShuf) among 63 array electrodes, and randomly generated trains (Rand), in which six different trains were generated by a computer, were also analyzed. Roughly speaking, there were two types of codes: high-appearance codes ranked up to code 21 and low-appearance codes starting at code 22 and beyond. The former were codes with three bits (“1”), including both end bits, whereas the latter were codes with four bits or more.

The appearance of codes that were often significant compared with those with shuffled or random trains was observed. For example, for code number 3 (“1101”), the number of appearances in the original (Org) train was significantly larger than that in the Shuf. Note that some codes were included within another code such as code number 5 (“10101”) that was also detected as code number 1 (“111”) if it passed the bit width test of 0.6–2.0 ms. Code number 5 (“10101”) was possibly counted twice; therefore, it had a protuberant peak.

Roughly speaking, we observed that the curve decreased according to the increase in code length. However, among the groups of the same code length, there was a mountain-like shape. This may have been because of the balance of the inner “1” position. Such codes [e.g., number 8 (“100101”)] with similar lengths of spaces (“0”) on the right and left appeared more than the unbalanced ones [e.g., number 7 (“100011”)].

## 3. Code Flow Map

**Figure 6:**
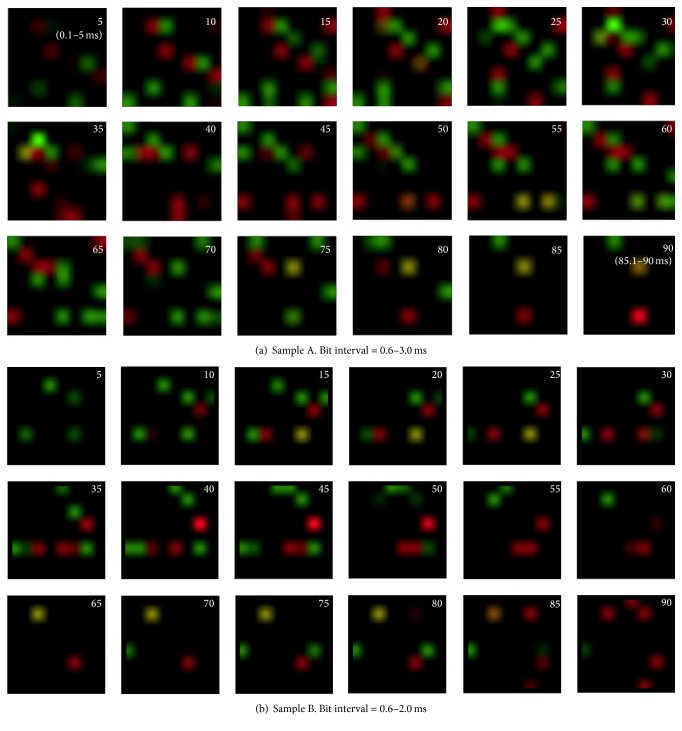
Code flow map for Samples A and B (Org). The serial images are from right to left and top to bottom, and “1011” and “1101” codes are expressed in red and green, respectively. Yellow indicates a mixed code. These spots are blurred to smoothen the movies. The frame interval is 5 ms and elapsed time is shown at upper right.

**Figure 7:**
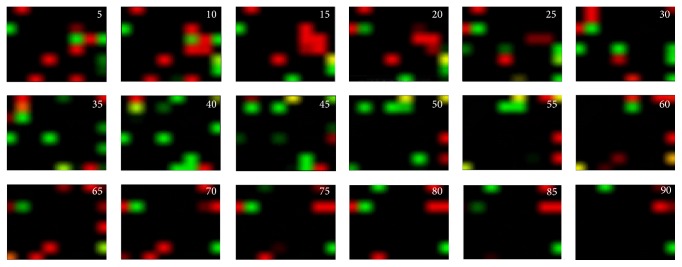
Code flow map, which is the same as that in Figure 6(a), but for a different trial. The appearance is noticeably different from that in Figure 6(a). The maximum brightness is normalized to 1 in the image.

**Figure 8:**
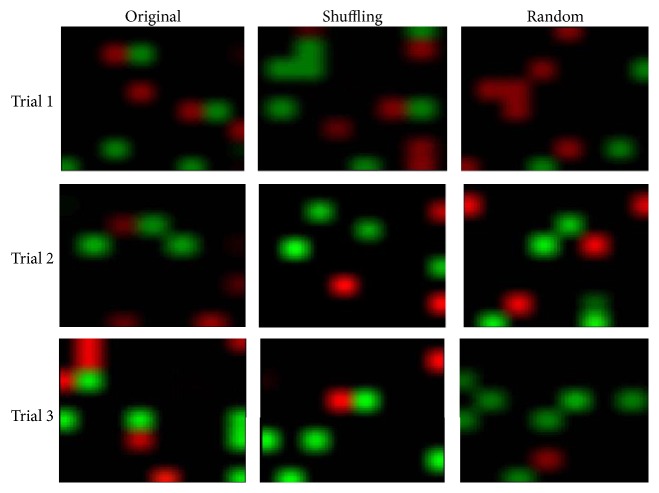
Movies in the three trials of code flow of Sample A. The code flows for the original, interval shuffled, and random spike trains in each trial are shown (http://www.nbl-technovator.jp/NBL_Tech/paper/CodeFlowFig8.pdf).

We can track the appearance of the codes as time-series images (Figure 2). Figures 6 and 7 show the time-series flow of “1011” and “1101.” These series are also shown as movies embedded in Figure 8 with AVI files. We found that the flow was stronger in the Org train than in the Shuf train. Although Figures 6(a) and 7 are from the same specimen as the successive experiments with several-minute intervals, the flow behaviors were markedly different. Within a short time range, such as 20 ms after stimulation, spikes in the different trials appeared at similar time instants, generating peaks on the PSTH [17]. In our case, however, observation time was too long, for example, more than several ten ms after the stimulation to show such synchronous or coherent behavior. That is, we deal with a time epoch where synchronization function by the stimulation has no effect such that each trial runs asynchronously and without repeatability. They were then analyzed statistically.

### 3.1. Quantitative Analysis of Flow

Let *f*(*E*, *C*, *F*) be a code-existing function, which represents in electrode *E* how much of the time code *C* exists in frame *F*. Here, a frame is composed of *N*_*F*_ = 50 bins, that is, 5 ms unless otherwise specified. The existing period is defined as that extending from the beginning bin of “1” to the ending bin of “1.” For example, if a code begins to appear at the middle of a frame period and continues to the next frame, then *f* = 0.5 (the code appeared in the half period of frame *F*). Thus, 0 ≤ *f*(*E*, *C*, *F*) ≤ 1 is usually true. However, if overlapping codes are detected, *f*(*E*, *C*, *F*) may be greater than 1. To avoid this, we normalized *f* by its effective root mean square (RMS) value so that

(1) $$\begin{array}{c} g\left(\;E,C,F\;\right)=\frac{f\left(E,C,F\right)}{{\text{RMS}}_{F^{\prime }\in \left\{1,2,\ldots ,N_{F}\right\}}\left[f\left(E,C,F^{\prime }\right)\right]}. \end{array}$$

Next, we calculated a local and instantaneous maximum cross-correlation in the next frame:

(2) $$\begin{array}{c} \varphi_{N}\left(\;E,C,F\;\right)=g\left(\;E,C,F\;\right)\cdot g_{N}\left(\;E_{\mathrm{Max}},C,F+1\;\right), \end{array}$$

where *E*_Max⁡_(*E*, *C*, *F*) = argmax_*E*′∈*N*(*E*)_ *g*(*E*′, *C*, *F* + 1) and *N*(*E*) is a set of 8 neighbors (8N) of *E* or a set of 20 neighbors (20N), where 8N is the 8 pixels around *E* (8 = 3 × 3 − 1) and 20N includes outside pixels adjacent to 8N, except for the 4 corners (20 = 5 × 5 − 1 − 4).

Next, we calculated the average Φ_*N*_(*C*) of *φ*_*N*_ over frame *F*, electrode *E*, and all the trials conducted for the same culture (each sample). Φ_*N*_(*C*) is the maximum cross-correlation between the current frame and the next frame, and *N* ∈ {8N, 20N}.

**Figure 9:**
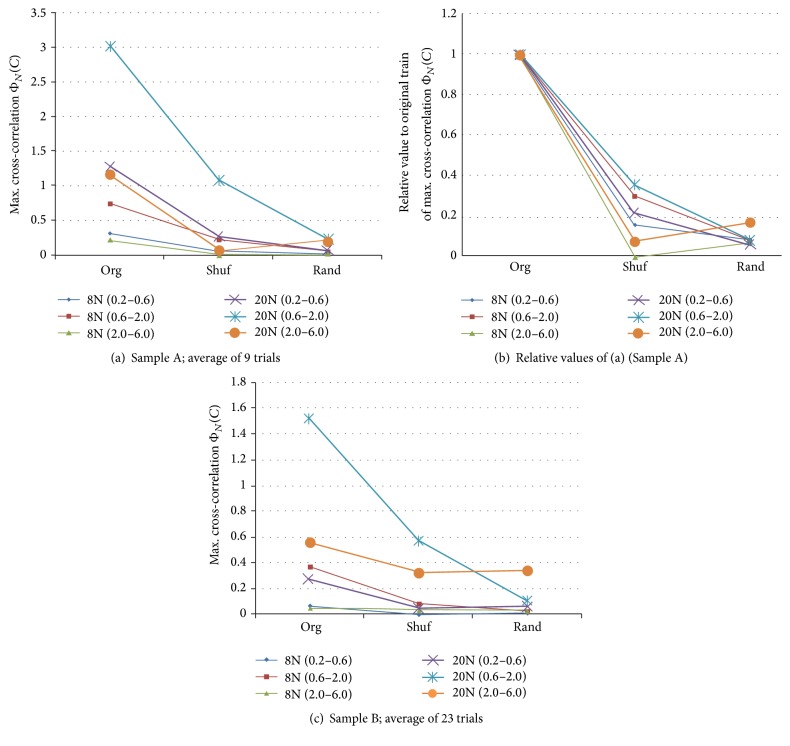
Maximum cross-correlation of a pixel with a 5 ms time difference (frame interval). For example, 20N (0.2–0.6) indicates that the “1011” (or “1101”) code with a 0.2–0.6 ms bit width had the maximum cross-correlation with some electrode within 20 neighbors in the next frame. The results are averages of “1011” and “1101.” From (b), we can see that the original train has clearly the code flow characteristic.

**Figure 10:**
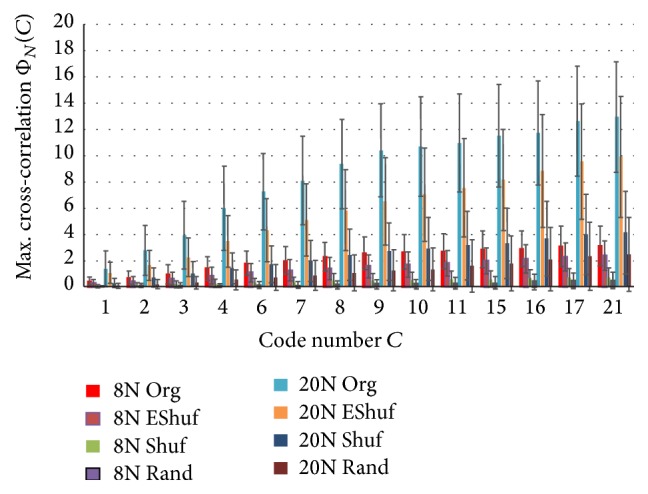
Maximum cross-correlation Φ_*N*_(*C*) in 8 neighbors (8N) and 20 neighbors (20N) for 14 major codes of Sample A. The frame width is 2 ms, and the bit width is between 0.6 and 2.0 ms.

**Figure 11:**
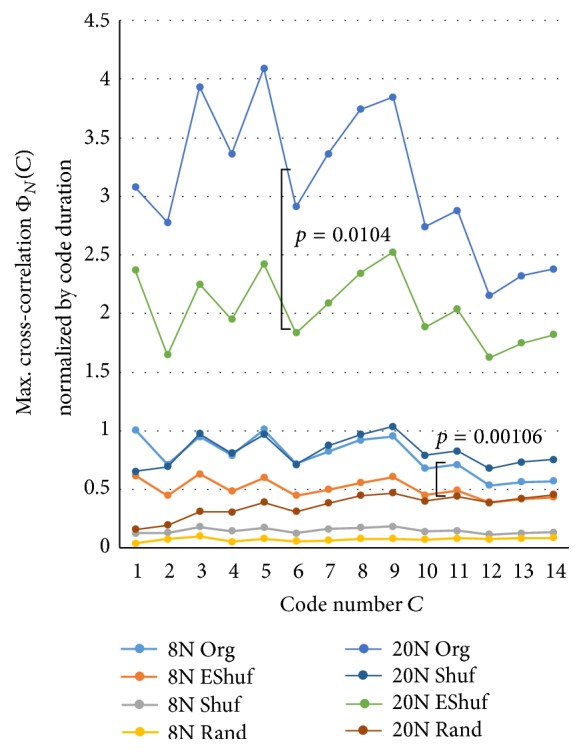
Maximum cross-correlation Φ_*N*_(*C*) that is normalized by the code duration for 14 major codes of Sample A. The *p* values are calculated from the EShuf/Org ratios of each code. This graph can be considered as another spectrum concerning the flow property of major codes. We can see that the normalized spectrum has flat (white) characteristic. That is, each major code can be considered as relatively independent. Though the values of 20N itself are larger than 8N, flow property difference between the original train and shuffled one of 8N is significantly larger than that of 20N as seen from *p* value.

The results of calculating Φ_*N*_(*C*) are shown in Figure 9 for Samples A and B with changing bit width. We observe that codes with a bit width between 0.6 and 2.0 ms of “1011” and “1101” seemed to be flowing most actively.  Figure 10 shows Φ_*N*_(*C*) for the other major codes with a bit width between 0.6 and 2.0 ms of Sample A. Because such values of the raw Φ_*N*_(*C*) depended on the duration (= length – 1) of the code, we further normalized it by the square of “code duration/3” so as to make the code “1011” standard, the duration of which from the beginning “1” to the ending “1” was code-length – 1 (= 3).  Figure 11 shows such a normalized flow Φ_*N*_(*C*) for the major codes with a bit width between 0.6 and 2.0 ms in Sample A. We observed that these codes also flowed actively. We observed that the values of the normalized cross-correlations were almost flat. The jags of the curves were caused by normalization with the stepwise code length. Then, the ratios of the EShuf value and the Org value were calculated for each code, and *p* values were obtained for 14 major codes. These findings suggested that the flow of the Org codes was significantly higher than that of EShuf, Shuf, and Rand. Because the maximum values of 20N were sought from wider ranges of about 3 times (*≒*20/8) compared to 8N, 20N were also about 3 times larger than that of 8N. That is, statistically maximum values of random-like Φ_*N*_(*C*) from 20 points (20N) are larger than that of 8 points (8N). However, since variance of that of 20 points is also larger than that of 8 points, *p* value of 8N was far smaller than that of 20N, and it showed that the flow of 8N was more significant than 20N under the assumption that the pseudorandom codes were almost independent. Further, as a noting parameter, we have *N*_*F*_ the time frame length or time difference calculating the cross-correlation. This should influence the value of cross-correlation according to the relationship with speed of spike transmission. That is, if the speed of spikes matches the distance (*μ*m) to 8N electrodes divided by *N*_*F*_ (ms), maximum cross-correlation of 8N becomes large, and vice versa in case of 20N. However, it is left to be examined in more detail.

The code can be regarded as a marker to track the flow of spikes. The codes seemed to move to other neighboring electrodes, first to 8N and then to 20N, while keeping the shape of the code. This was a stochastic phenomenon and therefore should be measured statistically as above.

### 3.2. Cross Analysis between Codes

To investigate the appearances of the codes, we extended the maximum cross-correlation in the next frame Φ_*N*_(*C*) to Φ_*N*_(*C*, *C*′) from code *C* to *C*′.  Table 1 shows the Φ_*N*_(*C*, *C*′) for Sample A. Because each entry was obtained by selecting the maximum direction, some exceeded 1. However, in the Φ_*N*_(*C*) case, it was normalized with the relative code duration itself to “1101” (i.e., 3), assuming that the same code appeared in the adjacent electrode at almost the same time coherently. In the Φ_*N*_(*C*, *C*′) case, we normalized it with the product of the relative code durations of *C* and *C*′ because each code duration was different and appeared randomly. Φ_*N*_(*C*, *C*′) had directionality and was therefore nonsymmetric. Next, we further normalized it so that the first average of each column became 1 and then each row became 1. We then further normalized it with the square root of the product of the corresponding diagonal components. Then, we obtained a matrix with diagonal components that were 1, as shown in Table 2. If each code is generated randomly, the nondiagonal component will become 1. If the code moves to the adjacent electrode exclusively, it will become lower than 1. In Sample A, the number of entries less than 1 was 119 among 182 nondiagonal entries. The *p* value of the hypothesis that the nondiagonal cross component is less than 1 was 0.00071. For Sample B, they were 136/182, and *p* = 1.81 × 10^−7^. For Sample C, they were 107/182, and *p* = 0.046. Furthermore, for the six generated Rand trains of Sample A, it was 90/182, and the *p* value of the hypothesis that the nondiagonal cross component of Sample A is less than that of Rand trains became 0.00092. This indicated that there was no bias in deriving and using this number of nondiagonal entries. This showed that the code had a tendency to flow as it was without changing its shape.

## 4. Discussion and Conclusion

To date, the coding mechanisms of neural networks have not been solved.

We observed spike trains that were produced by one-shot electrical stimulation of neuronal networks cultured on 8 × 8 multielectrodes. Each electrode accepted spikes from several neurons. We extracted short codes from each electrode and obtained a code spectrum. These codes were considered to be composed of the neuron circuits around the corresponding electrode. However, some codes may be observed by chance. To clarify this, we constructed code flow maps as movies of the electrode array to observe the code flows of “1101” and “1011.” They seemed to flow from electrode to neighboring electrode while keeping their shapes to some extent. We showed that if we shuffled the spike train interval, they became random with no flow.

To quantify the flow, we calculated the maximum cross-correlations of the codes with lengths less than 8. We found that the normalized cross-correlations were almost constant, irrespective of code. Furthermore, we showed that if we shuffled the spike trains in interval orders or in electrodes, they became significantly small.

Thus, the analysis suggested that the local codes around the electrode flow maintained the code shape to some extent, and they transported the information in the neural network. Since the bit width of the code was taken less than the refractory period, each spike composing code is considered from different neurons even without spike sorting. The short code may have been generated by local circuits, including feedback loops [14] or various transmission delays [11]. If so, the result will help to estimate the local circuit shape around the electrode. The analysis proposed here can also be regarded as the code decomposition of random-like spike trains with non-fully independent and semiorthogonal components (codes). Further, the codes can work as visible and trackable marks of the spike wave.

The problem is that the observed code maps have no repeatability except for the statistical characteristics as treated here or within such short term as 20 ms where poststimulus time histogram (PSTH) can be observed with coherency between neighboring neurons. For example, we can see “1101” like code shape PSTH in [17–19]. This short term coherency seems enough for such neuronal network where various information is flowing. The fluctuation of parameters of each neuron is inevitable and may work as perturbation element to define more suitable boundary as support vector machine [20]. Simulation of the code spectrum shown in this paper including issue of fluctuations as a macromodel will be discussed in another papers [16, 21].

The aim of our research is to challenge to elucidate communication function between remote positions of the brain [22, 23] with keeping correspondence between experimental findings and simulations, though there are difficulties of technological gaps between them. Information flow shown in this paper will give a base of communication function which needs identification or pattern classification function of spike waves and will give important base of brain intelligence [16].

Note that, in the communication, each neuron can receive not whole spike wave but spike train including the codes. Though depending on the perturbation level (SN ratio), we have already obtained recognition rate of more than 98% via partial spatiotemporal patterns of spike wave or spatially combined codes which are also spatiotemporal patterns. The results shown in this paper are preliminary step toward the final aim of elucidating mechanism of natural intelligence via communication link. These will be shown in our coming papers with still keeping correspondence between experimental findings and simulations.

In short, the aim of our research is to elucidate* computationally* the natural* intelligence* based on* neuroscience* experiments which is along by title of this journal and also should be the core target of it.

## Figures and Tables

**Table 1:** Maximum cross-correlation of Φ_*N*_(*C*, *C*′) to the next frame (*N*_*F*_ = 20), from the 14 major codes *C* to *C*′, and *N* = 8N. Bold indicates the diagonal entry.

	Code *C *→													
Code *C*′↓	**1.009 **	1.251	1.764	1.735	2.122	1.934	2.243	2.426	2.648	2.284	2.45	2.171	2.324	2.398
	0.588	**0.655 **	1.018	1.033	1.192	1.093	1.236	1.399	1.568	1.36	1.487	1.391	1.488	1.552
	0.561	0.781	**1.022 **	1.069	1.289	1.129	1.307	1.459	1.566	1.316	1.402	1.251	1.343	1.4
	0.576	0.711	0.971	**0.986 **	1.167	1.015	1.117	1.181	1.3	1.123	1.199	1.055	1.145	1.189
	0.315	0.378	0.551	0.628	**0.846 **	0.755	0.851	0.94	1.059	0.921	0.951	0.873	0.911	0.949
	0.255	0.287	0.415	0.512	0.614	**0.565 **	0.628	0.659	0.719	0.622	0.665	0.619	0.665	0.685
	0.372	0.456	0.651	0.666	0.783	0.695	**0.782 **	0.857	0.912	0.81	0.878	0.809	0.848	0.868
	0.281	0.43	0.653	0.651	0.775	0.695	0.822	**0.919 **	0.976	0.886	0.931	0.854	0.916	0.948
	0.219	0.254	0.372	0.377	0.445	0.442	0.476	0.532	**0.564 **	0.481	0.531	0.485	0.501	0.512
	0.135	0.211	0.291	0.27	0.331	0.293	0.332	0.391	0.433	**0.379 **	0.406	0.376	0.402	0.425
	0.209	0.242	0.263	0.266	0.316	0.327	0.372	0.409	0.443	0.393	**0.425 **	0.38	0.399	0.423
	0.117	0.162	0.207	0.191	0.237	0.223	0.269	0.305	0.334	0.302	0.325	**0.29 **	0.304	0.33
	0.18	0.206	0.29	0.33	0.386	0.35	0.393	0.428	0.453	0.427	0.463	0.41	**0.438 **	0.455
	0.056	0.096	0.161	0.179	0.241	0.222	0.249	0.305	0.316	0.278	0.311	0.278	0.292	**0.302 **

**Table 2:** Normalized maximum cross-correlation of Φ_*N*_(*C*, *C*′) to the next frame (*N*_*F*_ = 20) between the different codes *C* and *C*′of Table 1. The italic data shows values less than 1. By the normalization making the diagonal components 1, we can see more than half of the nondiagonal components are less than 1, and this means that statistically each code flows independently from other codes.

	Code *C*→													

Code *C*′↓	**1**	1.054	1.001	*0.928*	*0.944*	*0.946*	1.008	*0.965*	*0.986*	*0.962*	*0.969*	*0.932*	*0.946*	*0.901*
	1.056	**1**	1.047	1.001	*0.962*	*0.969*	1.007	1.009	1.058	1.038	1.066	1.082	1.098	1.057
	*0.959*	1.135	**1**	*0.986*	*0.99*	*0.952*	1.014	1.001	1.006	*0.956*	*0.957*	*0.926*	*0.943*	*0.907*
	1.083	1.136	1.044	**1**	*0.985*	*0.942*	*0.952*	*0.891*	*0.918*	*0.897*	*0.9*	*0.858*	*0.885*	*0.847*
	*0.83*	*0.847*	*0.831*	*0.892*	**1**	*0.981*	1.016	*0.993*	1.048	1.031	*0.999*	*0.996*	*0.985*	*0.947*
	*0.914*	*0.876*	*0.853*	*0.992*	*0.99*	**1**	1.022	*0.949*	*0.969*	*0.948*	*0.953*	*0.962*	*0.981*	*0.931*
	1.05	1.094	1.05	1.013	*0.992*	*0.967*	**1**	*0.97*	*0.966*	*0.971*	*0.988*	*0.988*	*0.983*	*0.928*
	*0.762*	*0.99*	1.012	*0.952*	*0.943*	*0.929*	1.011	**1**	*0.994*	1.021	1.007	1.001	1.02	*0.974*
	1.033	1.018	1.006	*0.961*	*0.943*	1.031	1.02	1.008	**1**	*0.965*	1.002	*0.992*	*0.972*	*0.917*
	*0.837*	1.114	1.036	*0.905*	*0.923*	*0.9*	*0.937*	*0.974*	1.01	**1**	1.007	1.011	1.027	1.001
	1.233	1.216	*0.889*	*0.848*	*0.837*	*0.951*	*0.996*	*0.968*	*0.982*	*0.985*	**1**	*0.97*	*0.968*	*0.946*
	*0.934*	1.096	*0.945*	*0.824*	*0.848*	*0.878*	*0.975*	*0.978*	1	1.023	1.033	**1**	*0.997*	*0.997*
	*0.998*	*0.974*	*0.921*	*0.989*	*0.963*	*0.959*	*0.99*	*0.954*	*0.944*	1.007	1.026	*0.984*	**1**	*0.958*
	*0.491*	*0.714*	*0.805*	*0.845*	*0.947*	*0.96*	*0.989*	1.07	1.039	1.033	1.086	1.053	1.05	**1**
